# Association between acute kidney injury and long-term mortality in patients with aneurysmal subarachnoid hemorrhage: A retrospective study

**DOI:** 10.3389/fneur.2022.864193

**Published:** 2022-09-01

**Authors:** Yangchun Xiao, Jun Wan, Yu Zhang, Xing Wang, Hanwen Zhou, Han Lai, Weelic Chong, Yang Hai, L. Dade Lunsford, Chao You, Shui Yu, Fang Fang

**Affiliations:** ^1^Department of Neurosurgery, Affiliated Hospital of Chengdu University, Chengdu, China; ^2^Key Laboratory of Pattern Recognition and Intelligent Information Processing, Institutions of Higher Education of Sichuan Province, Chengdu University, Chengdu, China; ^3^Department of Neurosurgery, West China Hospital, Sichuan University, Chengdu, China; ^4^West China School of Public Health, Sichuan University, Chengdu, China; ^5^Department of Nephrology, The First Affiliated Hospital of Chongqing Medical University, Chongqing, China; ^6^Department of Medical Oncology, Thomas Jefferson University, Philadelphia, PA, United States; ^7^Department of Radiology, Thomas Jefferson University, Philadelphia, PA, United States; ^8^Department of Neurosurgery, University of Pittsburgh Medical Center, Pittsburgh, PA, United States; ^9^Department of Neurosurgery, Dujiangyan People's Hospital, Dujiangyan, China

**Keywords:** intracranial aneurysm, subarachnoid hemorrhage, mortality, acute kidney injury, prognostic factors, complication

## Abstract

**Background:**

Though acute kidney injury (AKI) in the context of aneurysmal subarachnoid hemorrhage (aSAH) worsens short-term outcomes, its impact on long-term survival is unknown.

**Aim:**

We aimed to evaluate the association between long-term mortality and AKI during hospitalization for aSAH.

**Methods:**

This was a retrospective study of patients who survived >12 months after aSAH. All patients were evaluated at West China Hospital, Sichuan University, between December 2013 and June 2019. The minimum follow-up time was over 1 year. the maximum follow-up time was about 7.3 years. AKI was defined by the KDIGO (The Kidney Disease Improving Global Outcomes) guidelines, which stratifies patients into three stages of severity. The primary outcome was long-term mortality, which was analyzed with Kaplan-Meier curves and Cox proportional hazards models.

**Results:**

During this study period, 238 (9.2%) patients had AKI among 2,592 patients with aSAH. We confirmed that AKI during care for aSAH significantly increased long-term mortality (median 4.3 years of follow-up) and that risk increased with the severity of the kidney failure, with an adjusted hazard ratio (HR) of 2.08 (95% CI 1.49–2.89) for stage 1 AKI, 2.15 (95% CI 1.05–4.43) for stage 2 AKI, and 2.66 (95% CI 1.08–6.53) for stage 3 AKI compared with patients without AKI. Among patients with an AKI episode, those with renal recovery still had increased long-term mortality (HR 1.96; 95% CI 1.40–2.74) compared with patients without AKI but had better long-term outcomes than those without renal recovery (HR 0.51, 95% CI 0.27–0.97).

**Conclusions:**

Among 12-month survivors of aSAH, AKI during their initial hospitalization for aSAH was associated with increased long-term mortality, even for patients who had normal renal function at the time of hospital discharge. Longer, multidisciplinary post-discharge follow-up may be warranted for these patients.

## Introduction

Aneurysmal subarachnoid hemorrhage (aSAH) is a serious disease with high case fatality. Regarding short-term mortality, a meta-analysis of 33 studies found a case fatality from 8.3 to 66.7% and has decreased by 17% during the past three decades (1). This improvement in short-term survival underscores the need to consider the long-term outcomes. Patients who are alive 12 months after aSAH still face a 1.5-fold higher long-term mortality than the general population, mostly related to cardiovascular and cerebrovascular disease (2, 3). Moreover, the long-term effects of certain complications during hospitalization for aSAH are still unclear, leading to questions about best clinical practices and allocation of healthcare resources (2, 4).

Acute kidney injury (AKI) is a complication that has been reported as occurring in ~20% of patients who suffer strokes of any type (5, 6). Although the incidence of AKI has increased in the past decades, short-term mortality has declined because of advances in recognition and treatment, including the use of short term dialysis (7). Despite recovery of an initial event of AKI, patients remain at risk for the development of chronic kidney disease, cardiovascular events, and reduced long term survival (8).

For patients with aSAH, previous studies have shown that AKI is associated with increased short-term mortality (9–12); however, no study has evaluated the effects of AKI on outcomes beyond 6 months. The potential impact of AKI on long-term survival in patients with aSAH is important to assess for two reasons. First, there are no systematic efforts in place to monitor renal function long term after recovery from AKI (13, 14). Second, if AKI in the context aSAH leads to increased later mortality, methods to reduce subsequent renal toxicity (e.g., dehydration, careful use of both iodinated, and MRI compatible contrast agents) would become increasingly important.

This study aimed to examine the association between AKI and long-term mortality in 12-months survivors with aSAH in a large, single-center cohort of patients, with death records extracted from a government-run registry. We also examined the association between AKI and in-hospital complications.

## Methods

### Study design

Our study was approved by the Ethics Committee of West China Hospital (No. 20191133). In this retrospective, single-center, cohort study, we compared long-term mortality in 12-months aSAH survivors who either had or did not have an episode of AKI during their hospital admission. Data were collected from the electronic health records of all patients admitted to the West China Hospital, Sichuan University between December 2013 and June 2019. Subsequent survival records were extracted from the Household Registration Administration System, a government-run provincial database in China which mandates updated mortality records on all patients, even those who have no follow-up after discharge from the hospital. The institutional review board of the ethics committee of West China Hospital approved the study and granted a waiver of informed consent. The study complied with the STROBE criteria (Strengthening the Reporting of Observational Studies in Epidemiology) (15).

### Patient selection

All patients who were alive ≥12 months after the index aSAH were included. aSAH was confirmed by preoperative neuroimaging, cerebrospinal fluid analysis, or the patient's neurosurgeon during a surgical procedure to clip the aneurysm. Participants were excluded if their subarachnoid bleed was related to trauma, rupture of an arteriovenous malformations, detection of a fusiform aneurysm, or had a previous aneurysm treated before the index hospitalization. We also excluded patients whose serum creatinine was not obtained within 24 h of their admission and patients with a history of chronic kidney disease. We also excluded patients whose household registration was not in Sichuan province or whose personal identification number was not found in the electronic medical record system, because that number is the only way to study survival by searching the Household Registration Administration System database.

### Definition of AKI

We adopted the definition of AKI as defined by The Kidney Disease Improving Global Outcomes (KDIGO) guidelines (16). Namely, the change in serum creatinine during hospitalization was compared with baseline serum creatinine: stage 1, >0.3 mg/dL (>26.4 μmol/L) or >1.5- to 2-fold increase; stage 2: >2- to 3-fold increase; and stage 3: >4.0 mg/dL (>353.6 μmol/L), >3-fold increase in serum creatinine, or a need for renal replacement therapy. Urinary output was not considered for the definition of the AKI in this study due to insufficient data. At the time of discharge renal failure recovery was evaluated by comparing the discharge serum creatinine to the baseline serum creatinine. In this study, renal recovery is defined as serum creatinine returning to 1.5-fold of baseline serum creatinine without the need for renal replacement therapy. If the discharge creatinine was >1.5-fold of baseline serum creatinine, persistent renal dysfunction was confirmed.

### Outcome measures

The primary outcome was long-term mortality, defined as all-cause mortality at the longest follow-up after 1 year. Secondary outcomes were in-hospital complications, including hydrocephalus, delayed cerebral ischemia, rebleeding, seizures, pneumonia, intracranial infection, urinary tract infection, and bloodstream infection.

Death records were extracted from the databases of the Household Registration Administration System with a censoring date of April 20, 2021. In China, every resident has a unique identification number. When a patient dies, the law requires that a death certificate must be entered in the household registration in the bureau of public security within 30 days. As the death certificate database is accurate and complete, the rate of loss to follow-up of this study was negligible, and event time or censoring time is known exactly.

### Statistical analysis

All analyses were done with R version 4.0.3 (Foundation for Statistical Computing) and SPSS version 26 (SPSS Inc). Continuous variables were reported as means (with standard deviation), and categorical variables were reported as counts (frequencies). A 2-sided *P* < 0.05 was considered significant. We used multiple imputation to impute all missing values, the mean was imputed.

We examined unadjusted overall survival using Kaplan-Meier analyses and using the log-rank test to determine significant differences between groups. Multivariable survival analyses were done using Cox proportional hazards models to determine the independent effect of AKI on overall survival, with data presented as adjusted hazard ratios (HR) and 95% CI. All baseline variables with a value of *p* < 0.20 in univariate analyses were included into multivariate analyses. We selected the explanatory variables based on covariates examined in prior studies and clinical expertise. We assessed the degree of multicollinearity among the various variables using variance inflation factor (VIF) analysis with a VIF threshold of 10.

We also performed a propensity score-matched analysis (4, 17). A propensity score was calculated based on the probability of a patient having AKI. This score was calculated using a logistic regression model with AKI as the outcome and patient factors as determinants (age, sex, hypertension, diabetes mellitus, smoking, alcohol use, Hunt and Hess grade, Fisher grade, aneurysm location, aneurysm size, external ventricular drain, and aneurysm treatment). The matching variables were based on covariates examined in prior studies and clinical expertise (10, 12, 18–20). This predicted probability was used as a score for one to four matching cases with the nearest neighbor matching with calipers of 0.20 standard deviation; a difference more than 0.10 is considered meaningful. Overall survival was compared between matched groups.

We conducted a sensitivity analysis to assess the association between AKI and in-hospital complications, including AKI, hydrocephalus, delayed cerebral ischemia, rebleeding, seizures, and hospital infection.

We used the *E*-value to measure the robustness of the association between AKI and long-term mortality for unmeasured or unadjusted confounding (21). *E*-values were computed with an online *E*-value calculator (https://www.evalue-calculator.com/) (22). Model assessment was conducted using C statistic and Hosmer-Lemeshow goodness-of-fit statistics.

## Results

The definition of the study population is shown in Supplementary Figure 1. There were 2,592 patients living 12 months after aSAH. Records confirmed that 238 (9.2%) had an episode of AKI during hospitalization: 197 (7.6%) had AKI stage 1, 27 (1.1%) had stage 2, and 14 (0.6%) had stage 3 (Table 1). VIF values in this study were each substantially lower than threshold. Older age, male sex, history of diabetes mellitus, history of hypertension, and severe hemorrhage (including higher Hunt and Hess grade and higher Fisher grade) were associated with an increased risk of AKI. In addition, patients with AKI were more likely to have complications (Supplementary Table 1). AKI resolved in 199 (83.6%) patients, however, 39 (16.4%) had persistent renal dysfunction at the time of hospital discharge.

**Table 1:** Baseline characteristics of the patients.

**Characteristics**	**No AKI**	**AKI Stage 1**	**AKI Stage 2**	**AKI Stage 3**	*p* ^*^	***p* ^y^**
	**(*n* = 2,354)**	**(*n* = 197)**	**(*n* = 27)**	**(*n* = 14)**		
**Demographics**						
Age, year	55.2 (11.8)	56.7 (11.4)	60.5 (13.0)	58.1 (12.6)	0.01	0.43
Female	1,565 (66.5%)	109 (55.3%)	16 (59.3%)	8 (57.1%)	0.001	0.01
**Smoking**						
Never	1,788 (76.0%)	140 (71.1%)	19 (70.4%)	12 (85.7%)	0.22	0.53
Current	444 (18.9%)	48 (24.4%)	6 (22.2%)	2 (14.3%)		
Ever	122 (5.2%)	9 (4.6%)	2 (7.4%)	0 (0%)		
Alcohol abuse	444 (18.9%)	35 (17.8%)	8 (29.6%)	2 (14.3%)	1.00	0.50
**Medical history**						
Hypertension	581 (24.7%)	60 (30.5%)	6 (22.2%)	7 (50.0%)	<0.05	0.05
Diabetes	121 (5.1%)	18 (9.1%)	2 (7.4%)	1 (7.1%)	0.03	0.12
Anterior circulation aneurysm	2,152 (91.4%)	153 (77.7%)	20 (74.1%)	12 (85.7%)	0.19	0.43
Size of aneurysm, cm	0.68 (0.51)	0.68(0.55)	0.59 (0.21)	0.71 (0.25)	0.87	0.84
Miss	539 (22.9%)	29 (14.7%)	5 (18.5%)	4 (28.6%)		
**Hunt and Hess grade**						
I	246 (10.5%)	11 (5.6%)	3 (11.1%)	0 (0%)	<0.001	<0.001
II	1,338 (56.8%)	75 (38.1%)	9 (33.3%)	3 (21.4%)		
III	574 (24.4%)	68 (34.5%)	9 (33.3%)	3 (21.4%)		
IV	178 (7.6%)	38 (19.3%)	6 (22.2%)	7 (50.0%)		
V	18 (0.8%)	5 (2.5%)	0 (0%)	1 (7.1%)		
**Fisher grade**						
I	119 (5.1%)	6 (3.0%)	0 (0%)	0 (0%)	0.01	0.12
II	410 (17.4%)	27 (13.7%)	2 (7.4%)	1 (7.1%)		
III	309 (13.1%)	26 (13.2%)	3 (11.1%)	1 (7.1%)		
IV	1,000 (42.5%)	104 (52.8%)	17 (63.0%)	7 (50.0%)		
Miss	516 (21.9%)	34 (17.3%)	5 (18.5%)	5 (35.7%)		
External ventricular drain	30 (1.3%)	12 (6.1%)	3 (11.1%)	1 (7.1%)	<0.001	<0.001
**Treatment aneurysms**						
Clip	1,550 (65.8%)	149 (75.6%)	15 (55.6%)	9 (64.3%)	0.02	0.01
Coil	315 (13.4%)	15 (7.6%)	2 (7.4%)	0 (0%)		
No treatment	489 (20.8%)	33 (16.8%)	10 (37.0%)	5 (35.7%)		

AKI, acute kidney injury.

* Comparing patients without AKI to all patients with AKI.

y Comparing patients within the three subgroups of AKI patients.

In the 2,592 patients living at least 12 months after their aSAH, the mean observation period was 4.3 years (range 1.0–7.3 years). During the total follow-up interval 278 (10.7%) patients died. Kaplan-Meier survival analysis demonstrated a significantly reduced survival in patients with AKI compared with no AKI (Supplementary Figure 2). Univariate Cox regression analysis found that AKI compared with no AKI was associated with higher risk of death (HR 2.95, 95% CI 2.21–3.94). Multivariate Cox regression analysis also identified AKI (adjusted HR 2.13, 95% CI 1.57–2.88), age, aneurysms size, Hunt and Hess grade, external ventricular drain, and aneurysm treatment as independent predictors of long-term mortality (Supplementary Table 2). The *E*-value of adjusted HR for long-term mortality in multivariate Cox regression analysis was 3.68, with a confidence interval of 2.52, suggesting that unmeasured confounders were unlikely to explain the entirety of the effect. The Hosmer-Lemeshow test demonstrated *P* = 0.42, and the C statistic was 0.78, indicating the discriminatory ability of this model was good, without departure from a good fit.

We also adjusted confounders using propensity score matching. After matching, these groups were balanced in all patients' characteristics (Supplementary Table 3), and the association between AKI and mortality was unchanged (adjusted HR 1.77, 95% CI 1.27–2.48; Supplementary Figure 3).

**Figure 1:**
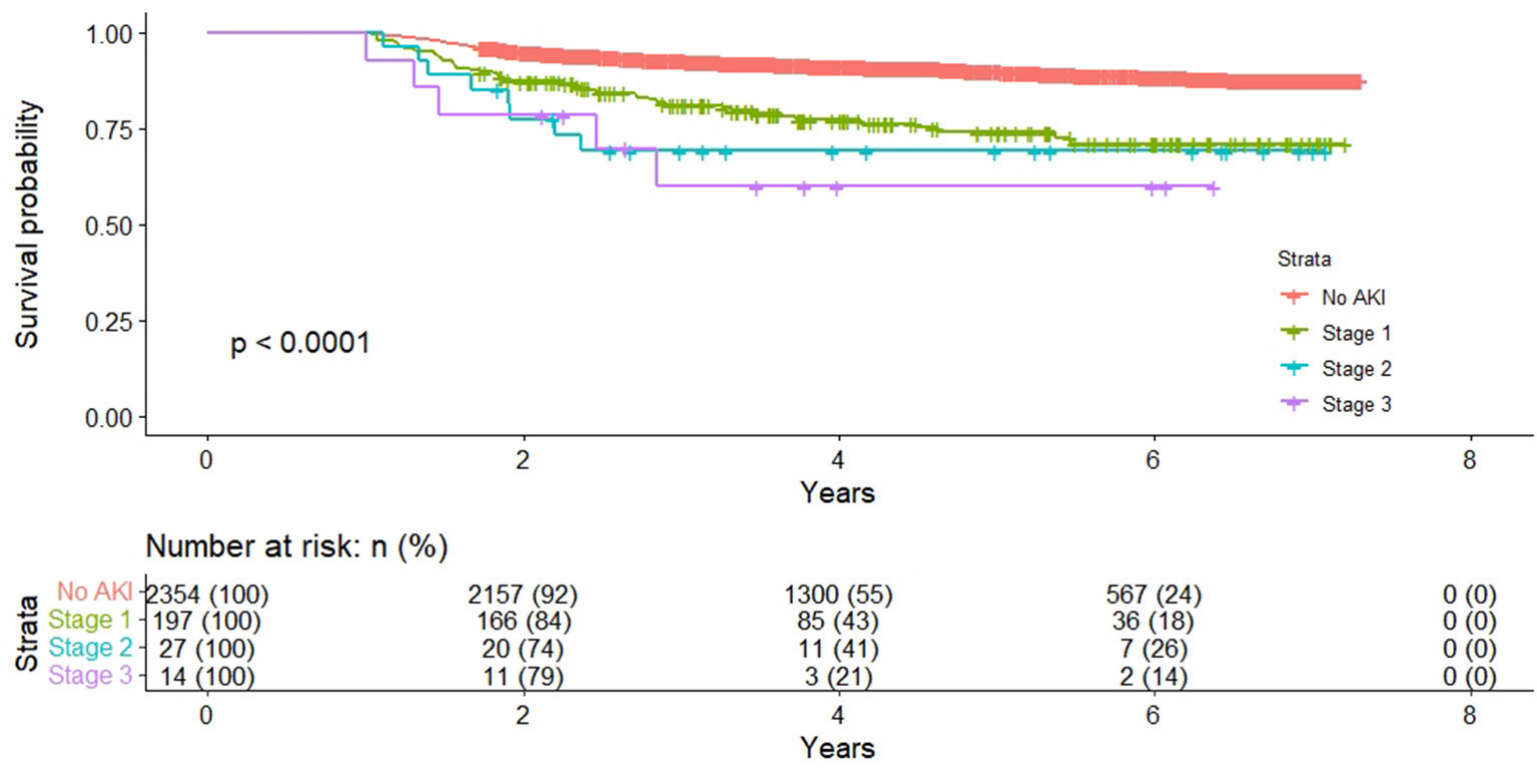
Long-term survival of patients who were alive 12 months stratified by acute kidney injury severity.

Kaplan-Meier survival curves illustrated that patients stratified by AKI severity still had significantly worse long-term survival over the follow-up period (Figure 1). The severity of AKI was associated with a progressively increased HR for death (Table 2). Compared with no AKI in multivariate Cox regression analysis, patients with stage 1 AKI had an adjusted HR of 2.08 (95% CI 1.49–2.89); patients with stage 2 AKI had an adjusted HR of 2.15 (95% CI 1.05–4.43), and patients with stage 3 AKI had an adjusted HR of 2.66 (95% CI 1.08–6.53).

**Table 2:** Cox proportional hazards model for long-term mortality, stratified by acute kidney injury severity.

**Acute kidney injury severity**	**Events, *n/N* (%)**	**Unadjusted**		**Multivariable adjustment**	
		**HR (95% CI)**	***p***	**HR (95% CI)**	***p***
No acute kidney injury	220/2354 (9.3)	[reference]		[reference]	
Stage 1	45/197 (22.8)	2.73 (1.98–3.76)	<0.001	2.08 (1.49–2.89)	<0.001
Stage 2	8/27(29.6)	3.68 (1.82–7.46)	<0.001	2.15 (1.05–4.43)	0.04
Stage 3	5/14(35.7)	5.03 (2.07–12.22)	<0.001	2.66 (1.08–6.53)	0.03

**Figure 2:**
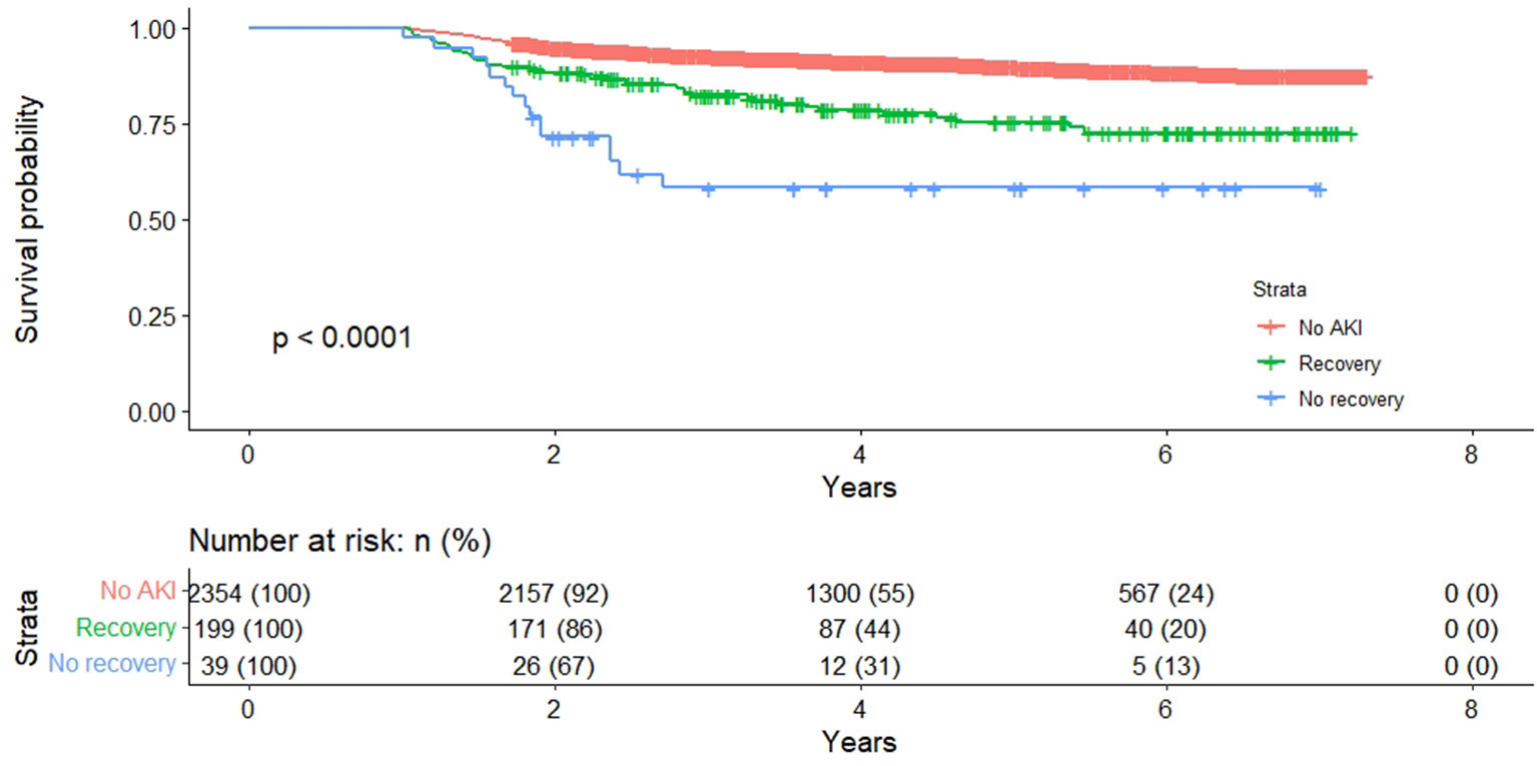
Long-term survival of patients who were alive 12 months with and without an acute kidney injury, stratified by renal recovery.

Patients with renal recovery at the time of hospital discharge had a significantly higher risk of dying later compared to patients without AKI (adjusted HR 1.96; 95% CI 1.40–2.74; Figure 2 and Supplementary Table 4). The survival rate for patients with AKI renal recovery at discharge was higher than patients without renal recovery (adjusted HR 1.95, 95% CI 1.04–3.67).

Effect modification is present with aneurysm location (*P* for interaction = 0.02) and aneurysm treatment (*P* for interaction <0.001; Supplementary Figure 4). There was no significant effect modification of AKI and long-term mortality on the other variables.

Sensitivity analysis remains a significant effect on AKI and long-term mortality (OR 2.01, 95% CI 1.35–2.96, *P* < 0.001; Supplementary Table 5).

## Discussion

In this large, single-center retrospective cohort study of patients who survived 12 months after aSAH, we found that AKI during hospitalization was associated with reduced long-term survival. As the severity of the AKI event increased, the risk of death progressively increased. Even patients who had recovered from AKI at the time of hospital discharge still had increased long-term mortality compared with patients without AKI.

### Comparison with other studies

To our knowledge, the association between AKI and long-term outcomes in patients with aSAH has not been studied previously; however, we are aware of several studies assessing short-term outcomes, which suggested increased mortality among patients with AKI (Supplementary Table 6). In a study of 787 patients with aSAH, patients with risk for renal failure as defined by RIFLE(Risk, Injury, Failure, Loss, and End stage) criteria, had worse 3-month outcomes and higher mortality rates compared to those not at risk (*P* < 0.0001) (12). In a claims database analysis of hospitalizations for aSAH in the United States, the incidence of acute renal failure (based on validated ICD-9-CM codes) in patients hospitalized for aSAH was 4.0%, and patients with acute renal failure had an increased likelihood of in-hospital death (OR, 2.14; 95% CI, 2.03–2.26) (10). In a recent study of 243 patients with SAH, AKI (defined by the AKIN [acute kidney injury network] criteria) was more frequent in ICU non-survivors but was not an independent predictor of ICU mortality in multivariable analysis (9).

### Mechanism

The underlying mechanisms of the relation between AKI and long-term mortality are still under investigation (8). AKI can be a reflection of the general insult of critical illness and may be a marker of systemic illness. Moreover, AKI can exhibit important effects on outcomes that extend well after hospital discharge. Several studies have identified that an episode of AKI may herald eventual long-term renal disease, such as recurrent AKI and development of chronic kidney disease (23, 24). Chronic kidney disease has been reported as 17.76 cases per 100 person-years following AKI (23). AKI also is associated with poorer long-term cardiovascular health. A systematic review of 25 studies involving 254,408 patients found that AKI is associated with a 58% increased risk of eventual heart failure and a 40% increased risk of acute myocardial infarction (25). Patients with chronic kidney disease and cardiovascular disease after AKI have higher mortality rates (8, 26).

### Strengths and limitations

This study has several strengths. The large cohort size allows adjustment for potential confounders and results in an enhanced dose-response relationship. We determined long-term mortality based on the household registration system that is considered accurate, up to date, and without lost follow-up.

This study has some limitations. First, potential weaknesses include the retrospective, observational analysis making causal inference difficult and subject to bias from unmeasured factors. Our hospital records could not systematically capture data on daily urine output values, which may lead to underestimation of the incidence of AKI. Second, this study did not contain information on either co-morbid diseases during the follow-up period or actual cause of death. Future studies should examine the association with cause-specific mortality. Third, our study is a single-center study. Whether our findings could be applied to other hospitals needed more research to confirm. Fourth, we cannot collect all confounders of the association between AKI and mortality, and thus other factors may affect the results. However, the *E*-value of the association suggested that unmeasured confounders were unlikely to explain the entirety of the effect.

### Implication

This study found that AKI during hospitalization for aSAH was associated with long-term mortality, even for patients with renal recovery at the time of discharge (13, 27). The current KDIGO guidelines recommend patients should be followed by a nephrologist for more than 3 months after an AKI episode in order to estimate kidney recovery and/or progression to chronic kidney disease (28). The present study indicates that patients should have long-term post-discharge monitoring of renal function, regardless of renal recovery at the time of discharge. AKI may result in ongoing progressive renal damage beyond the acute episode, despite marginal increases in serum creatinine. This is an important insight for all physicians who care for patients with aSAH, and future studies will need to determine the optimal timing for follow-up of renal function and develop biomarkers of the transition from AKI to chronic kidney disease for patients with AKI during hospital.

## Conclusions

In a large study of aSAH patients alive after 12 months, we demonstrated that AKI during hospitalization for aSAH was associated with increased all-cause long-term mortality. This risk was consistent with severity of AKI, and present during a median of 4.3 years of follow-up. Even for patients with renal recovery at the time of discharge, the risk of dying was higher than for patients with no AKI. Long-term mortality was highest in patients without renal recovery at the time of discharge.

## Data availability statement

The original contributions presented in the study are included in the article/Supplementary material, further inquiries can be directed to the corresponding author/s.

## Ethics statement

The studies involving human participants were reviewed and approved by the Ethics Committee of the West China Hospital (No. 20191133). Written informed consent from the patients/participants or patients/participants' legal guardian/next of kin was not required to participate in this study in accordance with the national legislation and the institutional requirements.

## Author contributions

Study concept: FF. Acquisition, analysis, or interpretation of data: YX, JW, XW, CY, SY, and YZ. Statistical analysis: YX and HZ. Drafting of the manuscript: YZ and WC. Design and critical revision of the manuscript for important intellectual content: All authors. All authors contributed to the article and approved the submitted version.

## Funding

This work is supported by the Key Laboratory of Pattern Recognition and Intelligent Information Processing, Institutions of Higher Education of Sichuan Province (Grant: MSSB-2019-11), National Key R&D Program of China (2018YFA0108604), the 1·3·5 project for disciplines of excellence-Clinical Research Incubation Project, West China Hospital, Sichuan University (21HXFH046), the innovation team project of Affiliated Hospital of Clinical Medicine College of Chengdu University (CDFYCX202203), the project of Sichuan Science and Technology Bureau (22ZDYF0798), and Clinical Incubation Program of West China Hospital, SCU (2018HXFU008).

## Conflict of interest

The authors declare that the research was conducted in the absence of any commercial or financial relationships that could be construed as a potential conflict of interest.

## Publisher's note

All claims expressed in this article are solely those of the authors and do not necessarily represent those of their affiliated organizations, or those of the publisher, the editors and the reviewers. Any product that may be evaluated in this article, or claim that may be made by its manufacturer, is not guaranteed or endorsed by the publisher.
