# Supplementary material for: Association between acute kidney injury and long-term mortality in patients with aneurysmal subarachnoid hemorrhage: A retrospective study
Source: Front Neurol. 2022 Sep 1;13:864193. doi: 10.3389/fneur.2022.864193 (PMC9475253; doi:10.3389/fneur.2022.864193)
Supplement: Supplementary file 1 [file Table_1.DOCX]

## Data Supplement

**eFigure 1. Flow chart of enrollment**

##### eFigure 2. Long-term survival of patients who were alive 12 months with and without an acute kidney injury during hospitalization before propensity score matching.

##### eFigure 3. Long-term survival of patients who were alive 12 months with and without an acute kidney injury during hospitalization after propensity score matching.

**eFigure 4. Subgroup Analysis of Association Between acute kidney injury and long-term mortality Table IV Multivariate Analysis for Predictors of in-hospital Mortality**

##### eTable 1. In-hospital Complications of patients with and without an acute kidney injury during hospitalization

##### eTable 2. Multivariate Analysis for long-term mortality

**eTable 3. Patient Characteristics with and without an acute kidney injury Before and After Propensity Score Matching**

**eTable 4. Cox Proportional Hazards Model for long-term Mortality, stratified by Renal outcome at the time of discharge Sensitivity analysis for long-term mortality**

**eTable 5. Sensitivity analysis for long-term mortality**

**eTable 6. The comparison with the other epidemiological studies**

#####
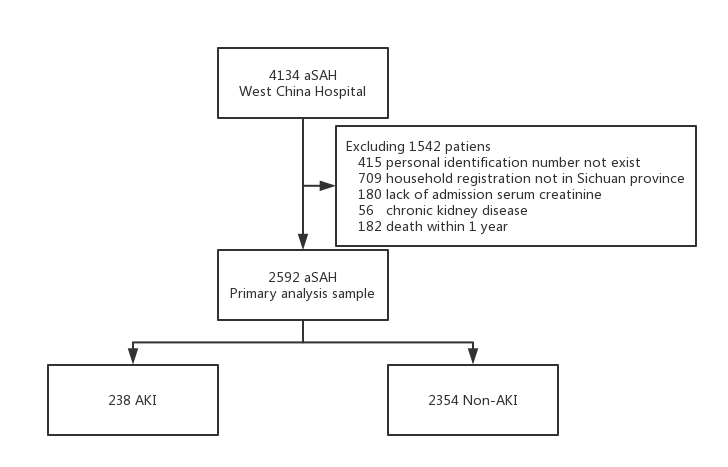
eFigure 1. Flow chart of enrollment

AKI: acute kidney injury

##### eFigure 2. Long-term survival of patients who were alive 12 months with and without an acute kidney injury during hospitalization before propensity score matching.


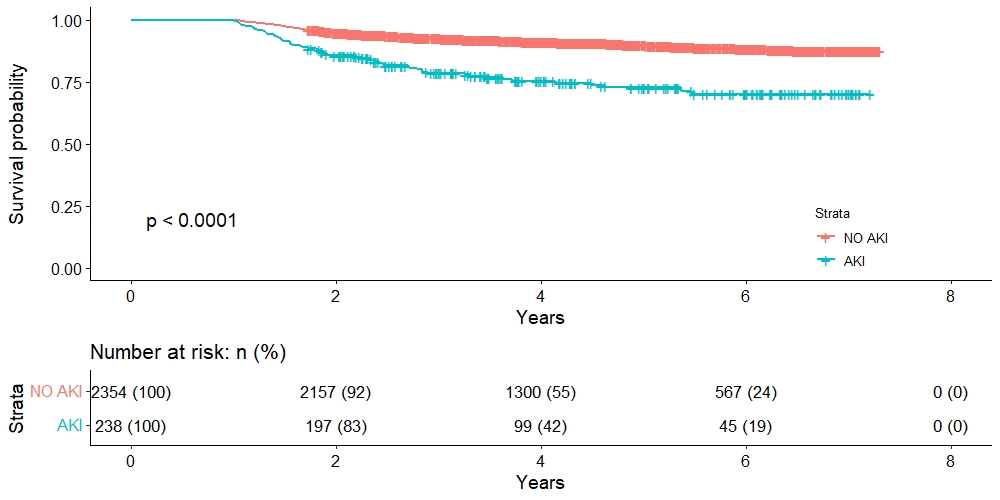


AKI: acute kidney injury

##### eFigure 3. Long-term survival of patients who were alive 12 months with and without an acute kidney injury during hospitalization after propensity score matching.


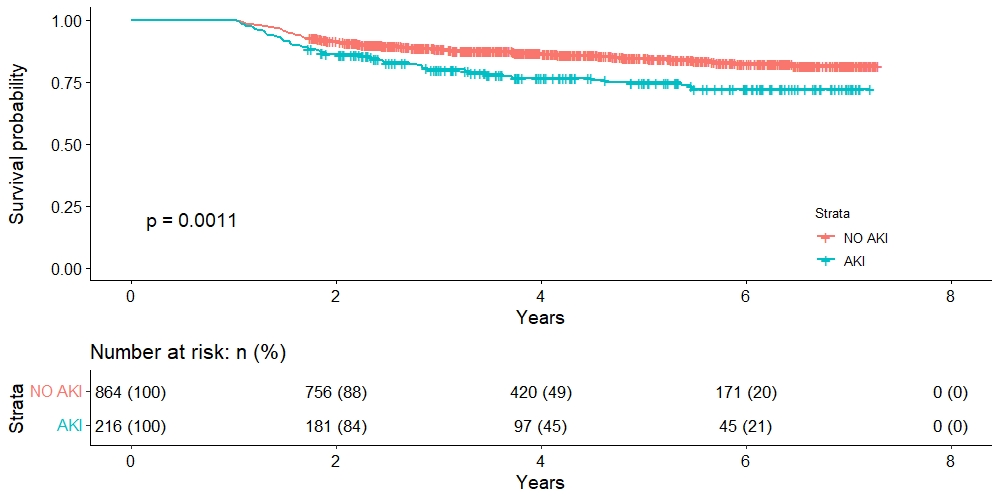


AKI: acute kidney injury

##### eFigure 4. Subgroup Analysis of Association Between acute kidney injury and long-term mortality

##### eTable 1. In-hospital Complications of patients with and without an acute kidney injury during hospitalization

| Outcomes | Unadjusted | | Multivariable Regression Adjustment | | Propensity Score Adjustment | |
| --- | --- | --- | --- | --- | --- | --- |
|  | OR (95% CI) | p | OR (95% CI) | p | OR (95% CI) | p Value |
| Hospital infection | 5.30(3.96-7.17) | <0.001 | 4.08(3.01-5.59) | <0.001 | 3.22(2.35-4.48) | <0.001 |
| Pneumonia | 5.06 (3.85-6.69) | <0.001 | 3.74(2.78-5.04) | <0.001 | 2.93(2.16-3.99) | <0.001 |
| Intracranial infection | 3.35(2.41-4.59) | <0.001 | 2.36(1.66-3.31) | <0.001 | 1.94(1.33-2.81) | <0.001 |
| Urinary tract infection | 2.73(2.00-3.68) | <0.001 | 2.30(1.67-3.14) | <0.001 | 2.34(1.65-3.30) | <0.001 |
| Bloodstream infection | 4.37(2.53-7.30) | <0.001 | 3.31(1.86-5.69) | <0.001 | 3.53(1.87-6.60) | <0.001 |
| Hydrocephalus | 2.22(1.51-3.19) | <0.001 | 1.31(0.84-1.97) | 0.22 | 1.33(0.85-2.02) | 0.19 |
| Re-bleeding | 4.67(3.02-7.10) | <0.001 | 3.59(2.27-5.57) | <0.001 | 3.24(1.96-5.31) | <0.001 |
| Delayed cerebral ischemia | 1.74(1.27-2.36) | <0.001 | 1.68(1.23-2.27) | <0.001 | 1.63(1.16-2.29) | 0.005 |
| Seizures | 2.31(1.23-4.07) | 0.005 | 1.87(0.97-3.40) | 0.05 | 2.15(0.99-4.46) | 0.04 |

##### eTable 2. Multivariate Analysis for long-term mortality

| Characteristics | Unadjusted | | Multivariable Regression Adjustment | |
| --- | --- | --- | --- | --- |
|  | HR (95% CI) | P | HR (95% CI) | P |
| Demographics |  |  |  |  |
| Age, year | 1.04(1.03-1.05) | <0.001 | 1.03(1.02-1.04) | <0.001 |
| Female | 1.04(0.81-1.33) | 0.77 |  |  |
| Current Smoking | 0.88(0.65-1.20) | 0.42 |  |  |
| Alcohol abuse | 0.73(0.53-1.02) | 0.07 | 0.71(0.50-1.00) | 0.05 |
| Medical history |  |  |  |  |
| Hypertension | 1.35(1.05-1.74) | 0.02 | 1.14(0.88-1.47) | 0.32 |
| Diabetes | 1.42(0.90-2.24) | 0.13 | 1.07(0.67-1.70) | 0.78 |
| Aneurysm characteristics |  |  |  |  |
| Anterior location | 0.97(0.75-1.25) | 0.8 |  |  |
| Size of aneurysm | 1.34(1.16-1.55) | <0.001 | 1.17(1.01-1.35) | 0.03 |
| Hemorrhagic characteristics |  |  |  |  |
| Hunt & Hess grade IV-V | 5.96(4.46-7.66) | <0.001 | 4.07(3.09-5.35) | <0.001 |
| Fisher grade III-IV | 1.47(1.13-1.91) | 0.004 | 1.27(0.96-1.70) | 0.10 |
| External ventricular drain | 4.79(3.00-7.63) | <0.001 | 2.44(1.50-3.99) | <0.001 |
| Treatment (clip or coil) | 0.38(0.30-0.46) | <0.001 | 0.31(0.25-0.40) | <0.001 |
| Acute kidney injury | 2.95(2.21-3.94) | <0.001 | 2.13(1.57-2.88) | <0.001 |

**eTable 3. Patient Characteristics with and without an acute kidney injury Before and After Propensity Score Matching**

| Characteristics | Before matching | | | After matching | | |
| --- | --- | --- | --- | --- | --- | --- |
|  | NO AKI (n=2345) | AKI (n=238) | SMD | NO AKI (n=856) | AKI (n=214) | SMD |
| Age | 55.18 (11.76) | 57.24 (11.70) | 0.18 | 57.51 (10.96) | 56.59 (11.58) | 0.08 |
| Female | 1565 (66.5) | 133 (55.9) | 0.22 | 505 (59.0) | 121 (56.5) | 0.05 |
| Current Smoking | 444 (18.9) | 56 (23.5) | 0.12 | 184 (21.5) | 47 (22.0) | 0.01 |
| Alcohol abuse | 444 (18.9) | 45 (18.9) | 0.001 | 156 (18.2) | 41 (19.2) | 0.02 |
| Hypertension | 581 (24.7) | 73 (30.7) | 0.13 | 252 (29.4) | 66 (30.8) | 0.03 |
| Diabetes | 121 (5.1) | 21 (8.8) | 0.15 | 63 (7.4) | 15 (7.0) | 0.01 |
| Anterior circulation aneurysm | 1757 (74.6) | 185 (77.7) | 0.11 | 652 (76.2) | 167 (78.0) | 0.05 |
| Size of aneurysm | 2.12 (2.71) | 1.67 (2.38) | 0.17 | 0.66 (0.45) | 0.67(0.54) | 0.01 |
| Hunt & Hess grade |  |  |  |  |  |  |
| I | 246 (10.5) | 14 (5.9) | 0.56 | 51 (6.0) | 14 (6.5) | 0.04 |
| II | 1338 (56.8) | 87 (36.6) |  | 349 (40.8) | 87 (40.7) |  |
| III | 574 (24.4) | 80 (33.6) |  | 295 (34.5) | 72 (33.6) |  |
| IV | 178 (7.6) | 51 (21.4) |  | 143 (16.7) | 37 (17.3) |  |
| V | 18 (0.8) | 6 (2.5) |  | 18 (2.1) | 4 (1.9) |  |
| Fisher grade |  |  |  |  |  |  |
| I | 119 (6.5) | 6 (3.1) | 0.27 | 21 (2.5) | 6 (2.8) | 0.07 |
| II | 410 (22.3) | 30 (15.5) |  | 103 (12.0) | 30 (14.0) |  |
| III | 309 (16.8) | 30 (15.5) |  | 117 (13.7) | 30 (14.0) |  |
| IV | 1000 (54.4) | 128 (66.0) |  | 449 (52.5) | 108 (50.5) |  |
| External ventricular drain | 30 (1.3) | 16 (6.7) | 0.28 | 20 (2.3) | 8 (3.7) | 0.08 |
| Aneurysm Treatment |  |  |  |  |  |  |
| clip | 1550 (65.8) | 173 (72.7) | 0.21 | 621 (72.5) | 155 (72.4) | 0.02 |
| coil | 315 (13.4) | 17 (7.1) |  | 63 (7.4) | 17 (7.9) |  |
| No treatment | 489 (20.8) | 48 (20.2) |  | 172 (20.1) | 42 (19.6) |  |

AKI: acute kidney injury; SMD: standardized mean difference

##### eTable 4. Cox Proportional Hazards Model for long-term Mortality, stratified by Renal outcome at the time of discharge

| Renal outcome at the time of discharge | Unadjusted | | Multivariable Adjustment | |
| --- | --- | --- | --- | --- |
|  | HR (95% CI) | p | HR (95% CI) | p |
| Renal recovery vs No acute kidney injury | | | | |
| No AKI | [reference] |  | [reference] |  |
| Renal recovery | 2.55(1.84-3.54) | <0.001 | 1.96(1.40-2.74) | <0.001 |
| No renal recovery | 5.35(3.17-9.03) | <0.001 | 2.89(1.68-4.97) | <0.001 |
| Renal recovery vs No renal recovery | | | | |
| No renal recovery | [reference] |  | [reference] |  |
| Renal recovery | 0.48(0.26-0.86) | 0.01 | 0.51(0.27-0.97) | 0.04 |

##### eTable 5. Sensitivity analysis for long-term mortality

| Characteristics | Multivariable Regression Adjustment | |
| --- | --- | --- |
|  | HR (95% CI) | P |
| Demographics |  |  |
| Age, year | 1.03(1.02-1.05) | <0.001 |
| Alcohol abuse | 0.67(0.45-0.98) | 0.045 |
| Hypertension | 1.16(0.86-1.56) | 0.34 |
| Aneurysm characteristics |  |  |
| Size of aneurysm | 1.34(1.02-1.65) | 0.008 |
| Hemorrhagic characteristics |  |  |
| Hunt & Hess grade IV-V | 4.35(3.05-6.17) | <0.001 |
| Fisher grade III-IV | 1.25(0.93-1.67) | 0.140 |
| External ventricular drain | 2.52(1.21-5.14) | <0.012 |
| Treatment (clip or coil) | 0.32(0.23-0.43) | <0.001 |
| Acute kidney injury | 2.01(1.35-2.96) | <0.001 |
| Hospital infection | 0.97(0.71-1.32) | 0.834 |
| Hydrocephalus | 1.99(1.33-2.92) | 0.001 |
| Re-bleeding | 1.74(1.01-2.89) | 0.034 |
| Delayed cerebral ischemia | 1.83(1.33-2.52) | <0.001 |
| Seizures | 2.16(1.23-3.95) | 0.016 |

##### eTable 6. The comparison with the other epidemiological studies

| Author | Year | Country | Design | Population | Definition | Incidence of AKI | Follow-up time | neurological outcome or mortality |
| --- | --- | --- | --- | --- | --- | --- | --- | --- |
| Zacharia et.al. [1] | 2009 | The United States | Prospective study | 787 aSAH patients | RIFLE criteria | 23.1% | 12 months | 3-Month mortality (OR 2.01). |
| Tujjar et.al.[2] | 2017 | Belgium | Retrospective study | 243 SAH patients | sustained oligoanuria or an increase in plasma creatinine | 12% | 28 d | poor functional outcome (17/93 vs. 8/109, P=0.03) |
| Rumalla and Mittal[3] | 2016 | The United States | Retrospective study | 260,885 aSAH patients | ICD-9 CM codes (584.5-584.9) | 4.0%. | Discharge | in-hospital mortality (OR: 2.14, 95% CI: 2.03-2.26). |
| Eagles[4] | 2019 | The United States | Secondary analysis of RCT | 413 aSAH patients | KDIGO criteria | 38% | 12 weeks | poor functional outcome (OR 1.40, 95% CI 0.83–2.34). |

AKI: Acute Kidney Injury

ARF: acute renal failure

RIFLE: Loss and End-Stage Kidney Disease

KDIGO; Kidney Disease: Improving Global Outcomes

References:

[1] B.E. Zacharia, A.F. Ducruet, Z.L. Hickman, B.T. Grobelny, L. Fernandez, J.M. Schmidt, R. Narula, L.N. Ko, M.E. Cohen, S.A. Mayer, and E.S. Connolly, Jr., Renal dysfunction as an independent predictor of outcome after aneurysmal subarachnoid hemorrhage: a single-center cohort study. Stroke 40 (2009) 2375-81.

[2] O. Tujjar, I. Belloni, J.M. Hougardy, S. Scolletta, J.L. Vincent, J. Creteur, and F.S. Taccone, Acute Kidney Injury After Subarachnoid Hemorrhage. J Neurosurg Anesthesiol 29 (2017) 140-149.

[3] K. Rumalla, and M.K. Mittal, Acute Renal Failure in Aneurysmal Subarachnoid Hemorrhage: Nationwide Analysis of Hospitalizations in the United States. World neurosurgery 91 (2016) 542-547.e6.

[4] M.E. Eagles, M.F. Powell, O.G.S. Ayling, M.K. Tso, and R.L. Macdonald, Acute kidney injury after aneurysmal subarachnoid hemorrhage and its effect on patient outcome: an exploratory analysis. Journal of neurosurgery (2019) 1-8.
